# Biotechnological applications of recombinant single-domain antibody fragments

**DOI:** 10.1186/1475-2859-10-44

**Published:** 2011-06-09

**Authors:** Ario de Marco

**Affiliations:** 1 University of Nova Gorica (UNG), Vipavska 13, PO Box 301-SI-5000, Rožna Dolina (Nova Gorica), Slovenia

## Abstract

**Background:**

Single-domain antibody fragments possess structural features, such as a small dimension, an elevated stability, and the singularity of recognizing epitopes non-accessible for conventional antibodies that make them interesting for several research and biotechnological applications.

**Results:**

The discovery of the single-domain antibody's potentials has stimulated their use in an increasing variety of fields. The rapid accumulation of articles describing new applications and further developments of established approaches has made it, therefore, necessary to update the previous reviews with a new and more complete summary of the topic.

**Conclusions:**

Beside the necessary task of updating, this work analyses in detail some applicative aspects of the single-domain antibodies that have been overseen in the past, such as their efficacy in affinity chromatography, as co-crystallization chaperones, protein aggregation controllers, enzyme activity tuners, and the specificities of the unconventional single-domain fragments.

## Introduction

Conventional poly-and monoclonal antibodies are still indispensable reagents in basic research and diagnostics. Nevertheless, both of them have some shortcomings-most of all the batch-to-batch variability of the polyclonal antibodies and the elevated costs and long time necessary for the production of the monoclonal ones. Furthermore, their dimension is detrimental for some diagnostic and therapeutic applications since it limits the efficient penetration into solid tumors and the passage through the blood-brain barrier. All these reasons urged the development of strategies aimed at the production of alternative scaffolds [1] and recombinant antibodies of smaller dimensions that could be easily selected, produced, and manipulated using standard molecular biology techniques.

Although a vast number of recombinant antibody structures has been proposed [2], the single chain (scFv) and the single domain (VHH, VH, and NAR V) formats are the most widespread for both research and industrial applications [2-4]. Recombinant antibodies seem particularly promising as immunoconjugates [5,6] and for activating the biosensor chip surfaces for detecting specific antigens [7]. Specifically, VHHs succeed in targeting brain epitopes by transmigrating through the blood-brain barrier [8,9], can be used for tuning and detecting the activity of cell proteins *in vivo *[10-13], provide better diffusion in fixed cells in comparison to conventional antibodies [14], and can simplify the generation of anti-idiotypic antibodies suitable for vaccination [15].

The success of the technology makes it impossible to review the entire literature concerning the different classes of antibody fragments and their applications in biology. The modes of application of VHHs, even though recently reviewed [4,16], constantly increase in numbers with the publication of new reports describing innovative uses. Therefore, this paper will on the one side integrate information concerning the established biotechnological utility of single-domain antibodies with the most recent data. On the other side, it will overview the potentials of the different classes of single-domain antibodies that developed massively in the last two years, such as immunoaffinity, assisted crystallography, protein aggregation and activity tuning, and toxin inactivation, for which a systematic update is now necessary.

### Unconventional single-domain antibodies

**Figure 1:**
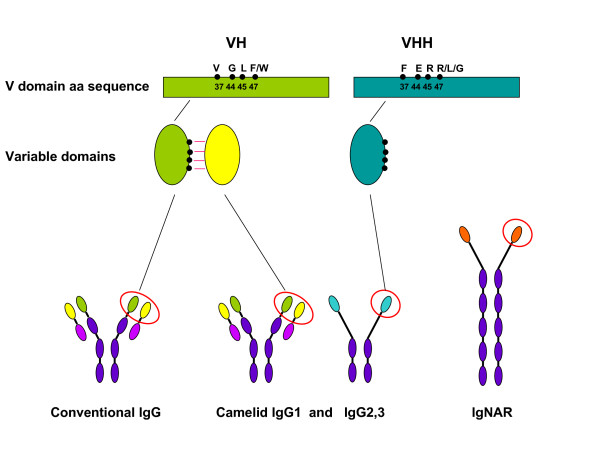
**Representation of the single-domain antibody specific features**. Single-domain antibodies can derive from different immunoglobulines. Camelid VHs and VHHs differ for four hallmark amino acid residues that are crucial for preserving the V domain stability when it is expressed paired to the VL domain or alone, respectively.

*Camelidae *single-chain antibodies and their recombinant VHH domains have ceased to be an exotic group with a merely theoretical interest and have become an established tool in biology and biotechnology. The number of publications that increases year in and year out testifies that an ever larger number of research groups work successfully with these molecules in different fields. Although not equally widespread, also the single chain antibodies of shark origin (IgNAR) have seen an increasing interest as a source of single-domain antibodies. In contrast, the scientific community is probably less aware of the qualities of other single-domain antibodies, the existence of which was for long time considered a simple anomaly (Figure 1).

VHH stability has been attributed to the mutation of the hydrophobic residues responsible for the interaction between VL and VH domains [17]. However, there are single-domains, such as the human VH domain HEL4, that conserve conventional VH hallmarks but are stable even in the absence of the pairing VL partner. HEL4 crystal structure suggests that the reason of its stability is due to an interaction by which the CDR1 partially masks the hydrophobic residues otherwise exposed on the framework region [18]. This structural arrangement resembles the stabilizing role of CDR3 residues observed in some VHHs, resulting in a loop partially bending over the framework [19] and indicates that single-domain antibodies can display different conformational patterns to prevent the exposure of potentially destabilizing amino acids. Although useful, the necessity to renounce the loop flexibility that contributes to the framework stability constrains the theoretical variability of the paratope in terms of shape and residues. In this sense, a VH domain stabilized by the sole replacement of the hydrophobic residues involved in the VL interaction has been proposed since it would preserve the maximal structural adaptability of the CDR3 and offer an ideal scaffold for generating libraries with loops hypermutated with respect to both length and residue combination [17,20,21]. At the same time, these libraries derived from human VHs would represent a source for selecting binders with no immunogenic response and, therefore, of great therapeutic interest.

For this purpose, it is relevant that individual VHs not only have structural features similar to those of the VHHs in terms of thermal stability and refolding efficiency [22-24], but also that they are perfectly functional in terms of antigen-binding characteristics, as initially reported by Ward et al. [25]. The mouse VH domains isolated by panning against lysozyme had affinity in the 20 nM range for their antigen and the same binding affinity was calculated for other VH single-domain antibodies isolated successively from a semi-synthetic library [26]. Another single-domain antibody (clone V86) was successfully used in several immunotechniques [27] and its nature as a structurally independent domain was confirmed by the observation that its specificity and affinity for the antigen were almost completely lost when it was coupled with a VL domain for reconstituting a scFv.

Single-domains with VH signature were isolated also after panning of *Camelidae *libraries designed for sampling only VHHs and their presence was considered as the consequence of material contaminations during the amplification steps of the single-domain DNA [28,29]. However, these VHs were functional and stable, indicating that they possessed structural features of independent single-domain antibodies.

Finally, specific classes of VH domains have been identified recently both in llama and mouse for which the presence of the light chains is entirely optional since they can either form conventional or equally functional but single-domain antibodies [30,31]. The stability and functionality of independent VHs is maintained also when they are expressed as intrabodies [32,33].

The identification of stable VH domains has been recognized as a potential opportunity to widen the overall pool of functional single-domain antibodies. From this perspective, the protocol used for single-domain library preparation has been modified for obtaining mixed libraries containing the whole repertoire of both VH and VHH domains. Stable and antigen-specific VH domains were isolated by panning from these libraries [34,35].

In contrast to VHs, only few independent VL domains have been reported as maintaining the binding capacity and specificity of the complete antibody molecule. These result from the *in vitro *cleavage of functional IgG fragments, as in the case of the VL binder specific for the streptococcal protein L [36] and of the VL-barnase immunotoxin that accumulates soluble and functional in *E. coli *cytoplasm [37].

### Targeting bacteria and phages

Antibodies directed against pathogenic bacterial-specific surface antigens enable to identify them in, and remove them from, biological samples. The advantage of using fragment antibodies lies in the fact that they lack the Fc domain, an IgG conserved region recognized by related proteins expressed by bacteria belonging to different species. Consequently, conventional antibodies are not suitable for specifically identifying a bacterial sub-group inside a complex population, whereas single-domain antibodies constitute a reliable tool for these diagnostic applications. For instance, single-domain isolated from a VH library [20] and directed towards the staphylococcal Protein A has been used in diagnostic applications based on both nanoparticles [38] and nanoaggregate-embedded beads [39].

Antibodies can be used for preventing *in vivo *the attachment of pathogenic bacteria to the target cells by competing with them for the host cell receptors [40,41]. The main limitation of such a strategy is that it is difficult to provide constantly the necessary amount of antibody because of the titer dilution and molecule clearance. The environment colonization with microbial cell factories that ensure the production and secretion of the competitor antibodies *in situ *represents a possibility to overcome this drawback, but IgG are not suitable for this application, in the way that antibody fragments are. Recombinant antibodies in scFv format raised against the streptococcal cell surface antigenI/II and transformed into lactobacilli were therefore used to infect the oral cavity of model rats to inhibit *Streptococcus mutans*, the agent responsible for dental caries. The method succeeded in decreasing the development of dental caries [42] and resulted more effective when antibody production was induced by a constitutive promoter [41]. Nevertheless, VHHs were successively preferred to scFvs because of their superior structural robustness and demonstrated their effectiveness in controlling the damage [43]. Orally administrated, transformed lactobacilli were also successfully used for delivering anti-TNF nanobodies to the colon at a concentration sufficient to significantly reduce chronic colitis [44].

The small dimensions of VHHs allow also their fusion to relatively large tags without negative effects in terms of recombinant yields. Therefore, their fusion to glucose oxidase for inducing the production of active forms of oxygen with microbicidal effect in the oral cavity has been proposed. This approach may offer a synergistic effect due to the contribution of the direct competition plus the antimicrobial enzymatic activity, but its effectiveness remains uncertain *in vivo *[43,45].

Mutagenesis is another tool that can be exploited to improve the *in vivo *reliability of antibacterial VHHs by selecting variants with superior features for a critical condition. A set of anti-*E. coli *F4 fimbriae antibodies was isolated and the most promising clone was further characterized. Even though very effective *in vitro*, it protected poorly the piglets from intestinal infection since it was rapidly degraded [40]. Therefore, its proteolytical stability was increased by mutagenesis for obtaining a significantly more effective binder suitable for oral treatment [46].

The control of the pathogenic bacterial infection can be also addressed by reducing the bacterial resistance to antibiotics. Microorganisms can survive in the presence of inhibitors since they produce enzymes that metabolize the antibiotics. This is the case of the beta-lactamase, a secreted enzyme that hydrolyzes ampicillin-like antibiotics. VHHs with beta-lactamase inhibitory effect have been isolated [47] demonstrating the feasibility of selecting single-domain antibodies that could silence this resistance strategy, although no biotechnological application has followed so far.

Unique features of the single-domain antibodies can be illustrated by a further example. The addition of recombinant VHH raised against a phage tail protein involved in the host recognition prevented the infection of *Lactococcus lactis *cultures by the p2 bacteriophage [48,49]. Subsequently, the lactobacilli have been engineered to produce directly phage-neutralizing VHHs at a rate sufficient for protecting themselves from infections [50]. This method represents an interesting approach for vaccinating microorganisms of biotechnological interest with the scope of protecting them from harmful aggressions of biotic and abiotic origin.

### Single-domain antibodies against fungi and protozoans

The potentials of single-domain antibodies have been only scarcely applied to fungi and protozoans. Nevertheless, those few projects have significantly contributed to the understanding of some general antibody structural features. It is the case of the antibodies raised against a cell wall protein of *Malassezia furfur*, a fungus implicated in dandruff. Since the selected VHH antibodies should be potentially included in a shampoo formulation, they had to resist to the harsh chemical conditions brought about by elevated concentrations of anionic and nonionic surfactants. Therefore, the panning washing conditions were adapted to represent the high-detergent content of shampoos [51]. This approach enabled the recovery of VHHs with specifically increased stability under denaturing conditions and to identify the key role of arginine at position 44 for improving this characteristic.

IgNARs, the single-chain antibodies of shark origin, have been used for identifying variable domains of the malarial Apical Membrane Antigen 1 of *Plasmodium falciparum *[52]. In sharks, IgNARs represent the antibody isotope responsible for antigen-driven immune response and the generation of high affinity antibodies as the consequence of rapid somatic hyper-mutation. Therefore, the authors simulated the natural mutational process by means of error prone PCR using conditions that introduced a single mutation in the whole sequence of the binder previously recovered by conventional panning. This approach allowed for the identification of more affine variants that differed from the original NAR V single-domain by having a less rigid structure, a feature that determined a better induced fit for the antibody-antigen complex and explained a slower dissociation rate of the mutants. Co-crystallization results indicated that the antibody's CDR3 loops penetrated deeply into hydrophobic clefts on the antigen surface and allowed for the identification of conserved residues that could be targeted preferentially in order to develop polymorphism-independent binders [53].

Single-domain antibodies can apparently also recognize effectively epitopes of parasite glycoproteins both in diagnostic settings and *in vivo*. It is the case of a VHH that binds the Ts14 protein of *Taenia solium *with sub-nanomolar affinity [54] and of other VHHs specific for oligomannose residues of the variant surface glycoprotein from *Tripanosoma b. rhodiense *[55-57]. Pan-reactive VHHs against *Tripanosoma *spp. were successively isolated for developing a rapid flow-cytometric diagnostic system for quantitative assess of the pathogen concentration in blood samples [57,58].

### Strategies for virus detection and neutralization

Also in the case of viruses, recombinant antibodies have been conceived as an inexpensive alternative to IgGs for masking the virion proteins involved in the host receptor binding and thus impairing the subsequent infection *in vivo *[59]. Single-domain antibodies possess a large protruding loop corresponding to the CDR3 and this structural feature represents an advantage over conventional antibodies in reaching the typically cryptic viral epitopes responsible for host recognition, similarly to what has been observed in the case of VHH competitive inhibition of the enzymatic catalytic site [60]. On the other hand, each virion usually contains tens of such epitope copies. Although it is true that conventional IgGs have no direct access to such hidden epitopes, their large dimension can increase virus neutralization by steric hindrance. To overcome the dimension limitation of VHHs and improve their neutralizing capacity *in vitro *and *in vivo*, their mass was increased by conjugation with immunoglobulins or inducing their N-glycosylation by providing suitable residues and successive expression in yeast [61-63]. The approach of defining the residues for N-glycosylation by mutagenesis should avoid specificity and affinity loss for the antigen due to paratope modification and reduced epitope accessibility. Nevertheless, the effectiveness of N-glycosylation for *in vivo *applications is still to be demonstrated because the large mannosylated carbohydrates synthetized by yeast are actively bound by liver receptors leading to fast VHH clearance.

The options for rendering single-domain fragments effective in human therapy described above are crucial in view of the fact that an increasing number of binders specific for viruses have been isolated, characterized, and successfully used *in vitro *and in animal models. For example, an effective diagnostic assay based on llama single-domains for identifying independently four different variants of the Marburg virus was delivered in three working weeks [64]. In this case, the availability of a semi-synthetic library enabled the reduction of the time necessary for the selection procedure, providing the evidence that reliable diagnostic immunoassays based on recombinant antibodies can be optimized during the initial phase of an infective outbreak.

VHHs selected using the group A rotavirus-conserved VP6 inner capsid protein are yet another example of efficacy of antibody fragments in neutralizing the virus and in protecting mice from diarrhea [65,66], where conventional antibodies had a modest effect. Using a strategy similar to that used for bacterial pathogens, VHHs raised against rotavirus were expressed on the surface of lactobacilli and orally administrated in a mouse model [67,68]. Severity and duration of the disease were significantly shortened as a consequence of a decrease in the viral load. An increase in the variety and availability of expression cassettes for the secretion or surface display of VHH antibodies should boost this approach for the protection from virus and bacteria infections [68].

Furthermore, VHHs and NAR Vs resulted effective in detecting poliovirus and inhibiting its replication *in vitro *[69,70], in binding vaccinia virus [71], and in preventing the assembly and secretion of hepatitis B virions in cellular models by the expression of engineered NAR V and VHH fragments directed against the cellular endoplasmic reticulum [72-74].

The inhibition of viral secretion using conventional antibodies has not been attempted in mammals because of the technical complexity, but it is becoming feasible using VHHs expressed as intrabodies. They can inhibit the multimerization of the HIV-1 Rev protein by binding to the target protein, sequestering it in the cytoplasm, and impairing the HIV-1 replication [75]. The cell uptake of the same virus is impaired by the small drug AMD3100 that acts as an antagonist for the CD4-activated co-entry receptor CXCR4. Conventional antibodies with the same function had previously been selected with the aim of isolating receptor antagonists with longer half-life than AMD3100, but they did not block sufficiently the virus-receptor recognition. As an alternative, anti-CXCR4 VHHs have been selected by competitive elution with the natural binder CXCL12 and successively proved to compete with AMD3100. Such VHHs behave as competitive antagonists and potently inhibit the virus uptake [76]. The strategy aimed at inhibiting the HIV-1 infection using VHHs that recognize envelope proteins and neutralize the virions by competing with the main cellular receptor CD4 [77-79] is conceptually similar. Finally, nanobodies have been also used to bind intracellularly the multifunctional Mf1 domain of the IRF-1 tumor suppressor with the idea of regulating this viral infection-stimulated regulative protein [80].

Single-domain antibodies were also successfully used for preparing piezoimmunosensors for the detection of HIV-1 virions [81]. The possibility of displaying at high density and controlling the orientation of these binders on the sensor surfaces strongly increases the method sensitivity.

Finally, the engineering of single-domain antibodies into multivalent anti-viral molecules have been proved to increase the neutralizing action against and to reduce the infectivity of Synctyal, Rabies and influenza viruses [82,83]. The simultaneous targeting of different epitopes of the same virus seems a promising alternative to increased avidity provided by multivalent display of the same antibody [83].

### Toxin identification and detoxification

The use of single-domain antibodies for toxin identification and neutralization has increased exponentially in the last two years. Two are the main reasons for the success, namely the possibility to use pre-immune libraries and the opportunity to have a scaffold structurally very stable, but extremely suitable for engineering application-specific modification.

VHH stability is crucial when harsh processing conditions, such as elevated temperatures, high detergent and reducing agent concentrations, or denaturing conditions, are used [51,84-86]. Specifically, toxic prolamins can be effectively extracted for quantification only in the presence of a solvent containing ethanol, 2-mercaptoethanol and guanidinium chloride. Conventional antibodies do not withstand these denaturing conditions and, therefore, are not available for immunological test. On the contrary, it was possible to select solvent-compatible VHHs with increased stability due to an introduction of extra disulfide bonds that constrained the CDR3 flexibility [84]. N-glycosylation is another parameter that allows the antibody stability tuning and can improve the antibody effectiveness *in vivo*. Similarly to what has been observed in the case of anti-virus single-domain antibodies, N-glycosylation increased the neutralizing capacity of VHHs against the *E. coli *heat-labile toxin in a cell model. Such an effect was attributed to the larger mass of the antibody that blocks simultaneously more than one of the five receptor-binding sites present on each toxin molecule [63].

Parallel panning of one-pot libraries yielded single domain antibodies in both VHH and NAR V format specific for ricin, cholera toxin, and staphylococcal enterotoxin B [71,87,88]. These studies demonstrated that pre-immune libraries can be used for rapid generation of antibodies against a large number of harmful antigens and that the single-domain antibody stability is beneficial for increasing their shelf life in diagnostic applications. Furthermore, the troublesome low sensitivity of single domain antibodies in immunological tests due to their monovalency could be overcome by using phage-displayed and not purified antibodies. This method allows the signal amplification by detecting several copies of phage-coating proteins in every single antibody-toxin binding event [89]. Although conventional IgGs are used preferentially for capturing toxins in microflow cytometry for multiplexed detection [90] and in the preparation of metal nanoparticle resistors for cholera toxin detection [91], single-domain antibodies seem suitable for similar applications [92].

A very interesting application in which single-domain cannot be substituted by conventional antibodies has been recently reported [93]. *Salmonella *SpvB toxin is secreted directly from the bacteria into the host cell cytoplasm and, consequently, is not accessible for extracellular antibodies. However, VHHs raised against the toxin can be expressed and maintain their functionality and specificity as intrabodies. They block the toxin at a molar ratio of 1:1 and prevent its pathological consequences at cellular level.

VHHs outperformed conventional antibodies also when used for the preparation of bivalent and bispecific constructs used for the *in vivo *neutralization of the AahI'/AahII scorpion toxins [94-96]. Multivalency induced by VHH polymerization [97-99] was exploited also to increase the neutralizing capacity of antibodies against the *E. coli *verotoxin 1 [100], alpha-cobrotoxin [101], and *C. difficile *toxin A [102], whereas the reduced production costs and the high density at which VHH can be coupled to resin substrates made feasible the preparation of hemofiltration columns for removing toxin shock syndrome toxin 1 from plasma to alleviate staphylococcal-induced sepsis [103,104]. Also an unconventional mouse VH resulting from an incomplete scFv antibody has been isolated and characterized because of its anti-toxin potentialities. In particular, it appeared to be a functional anti-idiotypic antibody for the HM-1 killer toxin with a higher affinity than scFv antibodies recovered from the same library for the neutralizing monoclonal antibody [105].

Toxins such as ricin and botulinum neurotoxin are classified as bioweapons and have been the object of a particular attention. In a project aimed at isolating anti-ricin antibodies, the panning protocol was first optimized using the toxin immobilized on the surface of microspheres [106]. The isolated VHHs were as effective as conventional monoclonal antibodies in blocking the ricin biological activity [107], but were superior in terms of specificity [108].

The majority of the publications on the toxin topic concerns the *Clostridium botulinum *neurotoxin (BoNT) because it causes frequently fatal disease and there is no drug able to reverse the symptoms once the toxin has entered the neuron. The interest in developing single-domain antibodies against BoNT is primarily motivated by the stability of these molecules even when they are expressed in the cytoplasm as intrabodies, facilitating not only diagnostic [109-111], but also therapeutic applications. Flow cytometry sorting of pre-immune, yeast displayed antibodies in VHH format allowed the isolation of anti-BoNT single-domains against the toxin light chain that inhibited the toxin protease activity *in vitro *and had structural features compatible for their production in the host cell cytoplasm [85]. Single-domain antibodies that inhibit the toxin protease activity by occupying the enzymatic cleft were also recovered from a mixed VH/VHH library [35], whereas VHHs isolated from an immune library retained their toxin inhibitory properties also when expressed in mammalian neuronal cell cytosol [112].

### Hapten targeting

Small molecules are not expected to be targeted efficiently by single-domain antibodies since they posses a limited number of conformational epitopes suitable for recognition by protruding single-domain paratopes. Nevertheless, there are several examples showing that VHHs can be exploited for detecting haptens as different as herbicides, caffeine, mycotoxins, trinitrotoluene, steroids and therapeutic drugs [113-120] for this antibody class can interact with its targets adopting different binding patterns [121]. For instance, the CDR1 loop provides a strong interaction for the azo-dye Reactive Red 6 [122], CDR2 and a framework residue contribute to the binding of Reactive Red 1 [123], whereas a non-conventional, substrate-dependent dimerization mechanism is involved in the binding of caffeine and its metabolites [124]. The stability of single-domain antibodies expressed in the cytoplasm has been used to induce their cellular accumulation as an effective tool for neutralizing the mycotoxin 15-acetyldeoxynivaleol *in vivo *[119].

### Reagents for immunodetection, purification and bioseparation

As an affinity-based technique, immunopurification presents some theoretical advantages over chromatographic methods based on chemical and physical properties. It can simplify complex multi-step procedures to a single step protocol, reducing production costs and time. Consequently, it can improve yields and limit potential product degradation. Nevertheless, immunopurification performed with conventional antibodies often requests extreme elution conditions that can damage the purified product. The advantages offered by single-domain antibody fragments in immunochromatography have been demonstrated for the first time by Verheesen et al. [125]. Their monomeric nature facilitated the elution of the target protein, whereas their physical stability and effective refolding allowed the regeneration of the column under harsh cleaning conditions for more than 2000 times. Mild elution conditions are necessary to preserve product structure and activity. From this perspective, panning VHH libraries has resulted as being an optimal solution since binders can be selected not only based on their specificity, but also on their elution features. In this way, unstable proteins such as serum factor VIII and alpha-1 antitrypsin have been immunopurified successfully using VHH antibodies for their capture and elution at neutral pH [126,127]. In a comparative assay with longer antibody constructs and complete IgGs, affinity columns prepared with VHHs enabled higher yields, probably because of the higher density at which they are bound to the matrix [128]. Such VHH-based affinity columns have found their applications for both purifying specific components from heterogeneous material [103,129-131] and for depleting very abundant proteins from samples in which it is necessary to detect the variation of scarcely represented polypeptides. It is the case of the platforms developed for the removal of IgG and human serum albumin from plasma before processing the samples in proteomic assays [132] and of the nanotrap for the purification of GFP-fused proteins from cell homogenates [133,134]. VHH-based affinity chromatography resulted also as very effective in removing contaminants such as DNA and virus particles [135]. At the same time, VHHs specific for adeno-associated viruses allowed for the simplification of the purification protocol of these molecules from five to one or two steps and for doubling of the final yields [136,137]. Beside the published reports and filed patent applications, a further indication of the single-domain antibody reliability as reagents for immunopurification can be inferred by the list of VHH-based affinity resins developed by biotech companies [138,139].

The small dimension (15 KDa, 4 × 2.5 × 3 nm) of single-domain antibodies and the possibility to produce them as recombinant proteins fused to suitable tags make them inexpensive binders that can be easily oriented at high density on capture surfaces such as chips and biosensors or adapted to a large variety of applications such as purification, imaging, immunomicelle and liposome preparation, or immobilization on nanoparticle resistors for the detection of cancer markers and cholera toxin [81,92,128,140-146]. A promising utilization strategy considers the VHH labeling via the 6xHis tag with metastable (99m) technetium for application in single-photon emission computed tomography (SPECT), a noninvasive monitoring technique for observing the tumor response to therapy [147-152], but nanobodies have been effectively labeled also with (68)Ga for immuno-positron emission tomography (PET) [153]. In both cases, their elevated tissue penetration and rapid clearance allow for optimal high-contrast, specific localization *in vivo *in a time significantly shorter than that enabled by using conventional IgGs [145,154,155]. Finally, the use of single-domain antibodies double-tagged with gadolinium and near-infrared dye Cy5.5 allowed the simultaneous optical and magnetic resonance imaging of brain tumor vessels [156].

### Single-domain antibody fragments as crystallography chaperones and tools for studying protein aggregation and activity regulation

The possibility of using antibodies to stabilize the conformation of proteins that undergo crystallization trials has been recognized since long time [157-159]. Nevertheless, conventional antibodies and their fragments have limitations due to their bulky mass (IgG and Fab fragment) or low stability (scFv) and, therefore, other molecules have been proposed, such as affibodies, fibronectin, and DARPins [160-162]. Single-domain antibodies seem a logical alternative since they can be produced inexpensively in short time, have a strong binding capacity, reduced mass, demonstrated capacity of providing "induced fit" to the antigen-antibody complex, and they improve crystal packing and X-ray phasing [163-165]. Although VHHs are amenable for simple site-specific incorporation of SeMet into their scaffolds, the fact that co-crystallizing with a single-domain antibody improves the complementary anomalous dispersion data acquisition and gives an ideal template for molecular replacement represents a substantial advantage for structure resolution [163]. Furthermore, VHHs are very flexible in terms of interface shapes [121-124] and, therefore, can adapt to a large variety of epitopes and block intrinsically flexible regions of their antigens [166]. VHHs have also been added directly to the solubilized membrane fraction for binding to and stabilizing the nitric oxide reductase membrane target protein [167] during the purification that resulted in the recovery of the antigen-antibody complex. No condition giving rise to diffracting crystals were found with the enzyme alone, but crystals were obtained in the presence of the VHHs. It has also been demonstrated that VHHs and their corresponding antigens can be co-expressed recombinantly in the same host and co-purified using tags fused to the antibody sequence, simplifying the protocol for producing antigen-antibody complexes [140].

The VHH-dependent approaches led to the resolution of several structures in the last few years [79,167-171]. VHHs seem to favor the formation of crystals of several different forms and to accelerate in a dramatic way the crystallization of recalcitrant protein complexes [168-170]. In the case of the KREPA6 editosome protein in combination with the Nb5 and Nb15 VHHs, all crystal contacts are obtained by the single-domain co-chaperones [170], while the role of VHHs is mainly to keep the flexible loop connecting the N1 and N2 domains of GspD in a fixed conformation [168] and to form stabilizing layers between layers of EpsI:EpsJ heterodimers [169]. Furthermore, although CDR3 is usually the major component involved in the antigen binding and crystal chaperoning [172], a feature deriving probably from its structural flexibility [79], in some cases CDR2 and even framework 3 can become pivotal chaperoning elements [170].

In the case of a study of the G protein coupled β_2 _adrenoreceptor, the VHH characteristics were useful at different levels. Whereas the crystallization of the inactive state of different members belonging to such receptor class has been achieved, the efforts to obtain a crystal of the receptor active state were frustrated due to receptor instability in the absence of G proteins. On the other hand, the receptor-G protein complex is unstable in the presence of the detergent necessary for the receptor stabilization. Therefore, suitable agonists were necessary for this aim. A high-affinity VHH agonist of the G protein was successfully identified by panning, its binding to the receptor was stable, maintained it in its active form during the crystallization trial functioning as a substrate agonist, and allowed the structure resolution operating as a crystallization chaperone [171].

VHHs also proved as being particularly useful for studying the intermediates of aggregation-prone polypeptides such as in the case of prion protein [173] and of the amyloidogenic fragments [174]. Single domain antibodies of different origin enabled the elucidation of the variety of morphologically distinct Aβ aggregates, blocking their polymerization development into non-toxic intermediates, and the clarification of the plausible mechanism of amyloidogenic protein self-association [175-180]. Specifically, domain swapping appears as a plausible self-association mechanism for amyloidogenic β2-microglobulin. Protein dimers undergoing swapping unmask amyloidogenic sequences that fold into two-stranded antiparallel β-sheet. Self-association of the sheet β-strands can provide the elongation mechanism by which large intermolecular β-sheets are built [179]. The first X-ray crystallographic structure of the complete Aβ_18-41 _fragment has been obtained by an innovative approach that may be of general interest for the study of tricky short peptides. The sequence corresponding to the fragment has been cloned inside the CDR3 of an IgNAR V and the chimeric protein expressed [180]. In such a way, amyloidogenic oligomerization was allowed through the direct contact of the exposed Aβ fragments, but its uncontrolled development prevented by the formation of an IgNAR cage. Crystallographic data confirmed the formation of an Aβ fragment-mediated tight tetramer in which the fine molecular mechanisms of the interaction could be observed and described.

The structures inferred from crystallographic datasets were also useful for the understanding of the mechanism by which a nanobody constrained the dehydrofolate reductase enzyme in its occluded form by binding it in a region adjacent to the active site. Such allosteric control of the enzyme activity raised hyperbolic inhibition kinetics and induced a conformational rearrangement of the whole enzyme structure [181]. Structural data were also necessary to explain the allosteric inhibition mechanism of the botulinum neurotoxin induced by another VHH [85]. In this case, it was possible to recognize that the antibody epitope corresponded to the α-exosite of the neurotoxin. This information has practical potentials since it identifies a target for the development of inhibitory small-molecule drugs.

Sometimes, the VHH-dependent allosteric enzyme modulation becomes extremely complex as illustrated by the case of the interaction between single-domains and the nucleoside hydrolase from *Trypanosoma vivax *[182]. The same antibody inhibited the catalytic pathway, but increased the product release rate and showed inhibitory or simulating behavior according to the substrate affinity for the enzyme. Another example of a complex modulation has been provided by the anti-human protein kinase C for which two sets of VHHs were isolated by panning immune libraries. All the five isolated VHHs were specific exclusively for a single isozyme (PKCε), and surprisingly, whereas three of them increased the kinase activity in a concentration-dependent manner, the remaining two inhibited the isozyme [183]. Taken together, these data indicate that VHHs possess not solely of a large array of paratope surfaces but that, beside their known capacity to block the enzyme active sites [60,184], they have alternative binding mechanisms involving different structural regions that are suitable for tuning their antigen-binding activity.

### Technical considerations-Conclusions

The number of reports dealing with single-domain antibody applications in different fields clearly indicates that this class of immunoreagents represents an important tool for research as well as biotechnological uses (Table 1). Furthermore, the described examples show that single-domain antibodies are complementary rather than alternative with respect to conventional antibodies in the sense that perform functions not possible to accomplish with IgGs.

**Table 1:** Available literature relative to the different biotechnological applications analyzed in the review

Field of Application	References
Bacteria and Phages	20,38-41,43-50

Fungi and Protozoans	51-58

Viruses	60-83

Toxins	35,63,71,84-89,92-112

Haptens	113-124

Immunodetection, Immunopurification, Bioseparation	81,92,125-156

Crystallography, Aggregates, Enzyme regulation	53,60,79,85,140,163-183,200

However, there are several technical issues that still need to be defined in a systematic way, at the recombinant antibody panning level as well as at the level of binder production strategies. For instance, the praxis indicates that one-pot libraries can deliver highly affine and specific antibodies [29,85,113,185-187], although somatic maturation was considered essential to obtain reliable binders. Moreover, it has been emphasized that non-immune library dimensions and display system were crucial for obtaining valid recombinant antibodies, but *E. coli*, yeast and ribosome display, even though successful at simplifying the panning and screening steps by means of flow-cytometric clone separation [188-191], did not perform better than small phage display libraries and recently even minimal libraries have proved to be potent means of selecting lead antibodies [192]. For a long time, an elevated affinity has been considered an absolute necessity, but now we know that it is important for *in vitro *application, whereas it can correlate inversely with appropriate tumor penetration *in vivo *[193,194].

Finally, several applications in chromatography, bioseparation, crystallography, or therapy need large amounts of recombinant antibodies and their fusion derivates, but their production technology has not improved significantly, although innovations concerning their expression in both bacterial periplasm [195,196] and in eukaryotic systems have been introduced [65,197]. However, a breakthrough seems to have arrived now with the advent of a technology enabling the cytolasmic accumulation of disulfide bond-dependent proteins. This approach aims at inducing the disulfide bond formation by co-expressing a sulfhydryl oxidase instead of disrupting the reducing pathways in the cytosol [198,199] and resulted in yields of VHH-fusion constructs in the range of several tens of mg/L culture [200].

Given the excellent qualitative performance of single-domain antibodies and their advantages in terms of structural features, stability, and production costs, a question remains to be answered as to why it has taken almost twenty years for them to become largely popular in the scientific community. Probably two reasons are the main responsible for this slow technology acknowledgment. The pretty restrictive intellectual property policy introduced by the inventor institutions with the "Hamers patents" that first defined the field has been extremely successful in boosting the spin-off companies funded to exploit the discovery, but it has probably prevented other actors from participating in the development. The second reason concerns the fact that for a long time there was only a minimal availability of those application-friendly tools for research and diagnostics that are necessary for making new technologies appealing to non-specialist researchers [201]. With the original patents expiring in the next few years and the recent accessibility of new libraries, protocols, and vectors suitable for different immunoapplications, we expect an increase of the interest of both research and industry for this class of recombinant antibodies in the near future.

## Competing interests

The author declares that they have no competing interests.
